# Comparing DSM-5 pathological personality traits in eating disorder patients and healthy control subjects using PID-5: results of a pilot study

**DOI:** 10.1192/j.eurpsy.2024.262

**Published:** 2024-08-27

**Authors:** J. Biliczki, J. Bognár, D. B. Pólya, S. Somogyi, S. Sutori, X. Gonda, S. Hamvas, J. Réthelyi

**Affiliations:** ^1^Clinical Psychology; ^2^Pediatrics; ^3^Semmelweis University, Budapest, Hungary; ^4^Psychiatry and Psychotherapy; ^5^Pharmacodynamics, Semmelweis University, Budapest, Hungary

## Abstract

**Introduction:**

The presence of eating disorders is often associated with serious physical complications, self-destruction, and suicidal tendencies. Furthermore, eating disorders may often present as a symptom of or in comorbidity with personality disorders. In order to treat eating disorder patients successfully we need a more complex and individual approach taking into consideration the specific personality dysfunctions and traits present in the patient underlying symptomatic manifestations. Recently a paradigm shift in conceptualisation of personality disorders led to the introduction of a dimensional concept focusing on severity of dysfunction in both ICD-11 and DSM-5 in its Alternative Model for Personality Disorders (AMPD). IN addition, DSM-5 as part of AMPD also considers the presence of 5 domains of pathological personality traits including 25 facets. This more complex mapping of personality could aid understanding personality contributors to psychopathology not only in personality disorders and could aid treatment by providing targets for psychotherapy.

**Objectives:**

Our aim was to compare pathological personality traits according to DSM-5 AMPD in eating disorder patients, and matched healthy control subjects.

**Methods:**

We are launching a large project focusing on personality disorders. For this analysis we used the adult form of PID-5 to assess pathological personality traits along 5 domains and 25 facets in eating disorder patients and psychiatrically healthy controls. Data were analyzed with the Mann-Whitney test using R.

**Results:**

Preliminary results of a pilot analysis in 14 eating disorder patients and matched psychiatrically health controls are shown. Comparing the data of the two groups, a significant difference was observed in several personality facets, including Anxiousness, Deceitfulness, Grandiosity, Impulsivity, Manipulativeness, Perceptual dysregulation, Rigid perfectionism, Submissiveness, and Unusual beliefs. These differences in the above facets reflect differences in the two groups in all pathological personality domains including Anhedonia, Negative affect, Antagonism, Disinhibition, and Psychoticism.

**Conclusions:**

Our results show complex differences between eating disorder patients and healthy control subjects in several facets, pointing to a unique pattern and the affectedness of all pathological personality domains. Such results could possibly add to identifying personality trait targets for psychotherapy in eating disorders besides increasing our understanding on the etiopsychopathology of this serious psychiatric illness. Our study is ongoing, but more complex analyses involving further measures and variables in larger samples bring the hope for increasing effectiveness of treatment for anorexia.

**Disclosure of Interest:**

None Declared

